# Why Adolescents Choose to Stop or Never Start Vaping: Evidence From South Africa

**DOI:** 10.1093/ntr/ntaf266

**Published:** 2025-12-24

**Authors:** Asya van den Bosch, Samantha Filby, Maria Florence, Richard N van Zyl-Smit, Sebastian Kurten

**Affiliations:** Department of Interdisciplinary Social Sciences, Utrecht University, Utrecht, The Netherlands; Research Unit on the Economics of Excisable Products, School of Economics, University of Cape Town, Cape Town, South Africa; Department of Psychology, Faculty of Community and Health Sciences, University of Western Cape, Cape Town, South Africa; Division of Pulmonology and Department of Medicine, University of Cape Town and Groote Schuur Hospital, Cape Town, South Africa; Department of Interdisciplinary Social Sciences, Utrecht University, Utrecht, The Netherlands

## Abstract

**Introduction:**

E-cigarette use among adolescents is increasing rapidly, raising serious concerns about nicotine addiction, health risks, and long-term developmental impacts. However, limited research has explored adolescents’ motives for never starting or quitting vaping, specifically in the global south. Therefore, this article aims to examine adolescent high school learners’ motives for quitting or never starting vaping.

**Methods:**

This study uses qualitative data from a large cross-sectional survey conducted among 25 149 South African high school learners aged 13–19 (grades 8–12), to examine motives for quitting or never starting vaping. Among learners who quit or never started vaping, a total of 11 401 (5162 and 6239 respectively) usable open-ended responses were thematically analyzed.

**Results:**

Health and addiction concerns emerged among the most frequently mentioned motives for both never starting vaping, mentioned by 46.0% of learners who never vaped, and quitting vaping (33.5%). Lack of appeal was also commonly cited among learners who quit vaping, especially among older learners (44% of grade 12 learners). Additionally, some learners, particularly boys, referred to athletic performance and fitness as reasons for never starting (13.5%) or quitting (14.4%). Other motives, such as access and economic barriers were only cited by a small minority as reasons for vaping abstinence (2.6%) and cessation (4.7%). Differences in motives by gender and grade level were observed.

**Conclusions:**

These findings can inform the development of context-specific prevention and cessation strategies. Our findings indicate that implementing educational campaigns on the consequences of vaping and restricting access, for example, through increasing taxes on vaping products, might aid adolescent vaping prevention and cessation efforts.

**Implications:**

As adolescent e-cigarette use rises, gaps in knowledge to fuel intervention and prevention strategies remain. The prominence of health and addiction concerns highlights the importance of implementing awareness campaigns on the negative effects of vaping on health. With vaping losing appeal over time, reinforcing negative perceptions through ongoing education and health reminders might effectively support cessation efforts. Because participants did not cite economic barriers for not starting to vape, this suggests missed opportunities for economic measures such as higher taxes.

## Introduction

The usage of electronic cigarettes (e-cigarettes) has increased globally in recent years.^1^ These products are often marketed as an alternative to smoking or assisting with cigarette-smoking cessation in adults; however, vaping is increasingly initiated by teenagers who have never smoked, posing unwarranted health risks.^1^^,^^2^ This trend can also be found in South Africa: while the South Africa Demographic and Health Survey in 2016 estimated that only 2.9% of women aged 15–19 and 2% of men occasionally used vapes,^3^ in 2023, around 17% of South-African learners attending fee-paying high schools reported current e-cigarette use, with 36.7% reporting to have ever-used vapes, raising concerns about early nicotine addiction and the long-term public health implications.^4^

Urgent action is needed, as the rise of vaping has coincided with an increase in vaping-related lung injuries.^5^ Young people who vape report more respiratory symptoms, including wheezing, shortness of breath, and bronchitis-like symptoms.^6^ E-cigarette use may also be associated with higher prevalence and aggravation of asthma in youth.^2^ Nicotine-containing e-cigarettes harm adolescent brain development and cognition.^1^^,^^7^^,^^8^ Xie et al.^9^ found that exclusive e-cigarette users were three times more likely to report serious difficulty concentrating, remembering, and making decisions compared to individuals who never used e-cigarettes. Their study also found that this effect was more pronounced in e-cigarette users than in exclusive combustible cigarette users, underscoring the need for targeted prevention efforts among adolescents.

To develop effective prevention and intervention strategies that support adolescents in vaping non-initiation or cessation, their underlying motives have to be taken into consideration. Factors leading to vaping among South African learners such as social influence, enticing flavors, and stressors have been well documented.^4^ Even though there is research available on adult cigarette smoking cessation,^10^ there is a lack of large-scale research on the factors that drive vaping non-initiation or quitting among adolescents, especially in the global south. Given the rising prevalence of e-cigarette use in this age group, the need for evidenced-based, youth-centered prevention, and cessation programs is both timely and essential.

To bridge this gap, this research focuses on the South African context. While other countries, among which countries as Gambia, Mauritius, and Uganda have adopted laws regulating e-cigarette use, in South Africa e-cigarettes are sold freely to minors, marketing remains unchecked, and public vaping unregulated.^11^ A small excise tax of ZAR3.18 per milliliter e-liquid is active (as of 2025), which is however consistently below the tobacco tax.^11^ The lack of regulation is influencing South African adolescents' expectations of the safety of vaping.^12^ Health organizations such as the Cancer Association of South Africa are calling for more education and awareness efforts, improved access to cessation support, and for the implementation of the South African Tobacco Products and Electronic Delivery Systems Control Bill to be fast tracked.^13^^,^^14^

To aid the development of effective vaping prevention and cessation strategies, this work answers three key questions: (1) What motivates learners to never start vaping? (2) What motivates learners to quit vaping? (3) Do these motives differ by gender and school grade?

## Materials and Methods

This study draws on data collected between January and October 2023 in South Africa. A total of 25 149 high school learners, aged 13– 19, from grades 8 to 12 participated in a self-administered online survey. In our sample, 45.88% (*n* = 11 448) identified as female, and 51.72% (*n* = 12 906) identified as male, 2.40%, (*n* = 599) did not identify as male or female. Learners were recruited from 52 fee-paying schools across eight of South Africa’s nine provinces, using a snowball sampling approach. The number of schools was determined to detect a relative 10% difference in vaping incidence between sub-groups of interest, based on an estimated incidence rate of 15.5% from an initial pilot feasibility phase.^3^ Ethics approval was provided by the University of Cape Town Faculty of Health Sciences Human Research Ethics Committee (UCTHREC 248/2022).

Of the 25 149 respondents, 7753 (36.7%) reported ever using vapes. Of these, 5546 answered the open-ended question on why they stopped (“If you have tried vaping/smoking or used for a short time, why did you stop or decide to not continue?”), with 5162 usable responses. The full sample included 13 368 (53.2%) learners who had never vaped; 6638 of them answered the question on why they never vaped (“If you have never vaped/ smoked – please briefly explain why not”), and 6239 responses were usable. Responses were excluded if mocking, incoherent, or inconsistent.

## Results

Responses to the two open-ended questions on motives to never vape or to stop were manually coded using thematic analysis.^15^ Based on existing literature,^4^ two coders jointly developed a 15-code framework (Appendices S1 and S2, available on https://osf.io/9ap3d/). Both coders then independently assigned codes to 1000 responses. Intercoder reliability was acceptable (Kappa = 0.833). Afterwards, codes were further grouped into the following themes: rules, culture, and morality; unspecified negative consequences; access and economic barriers; lack of appeal; social influence; athleticism and fitness; and health and addiction concerns.

### Motives for Never Starting Vaping

**Figure 1:**
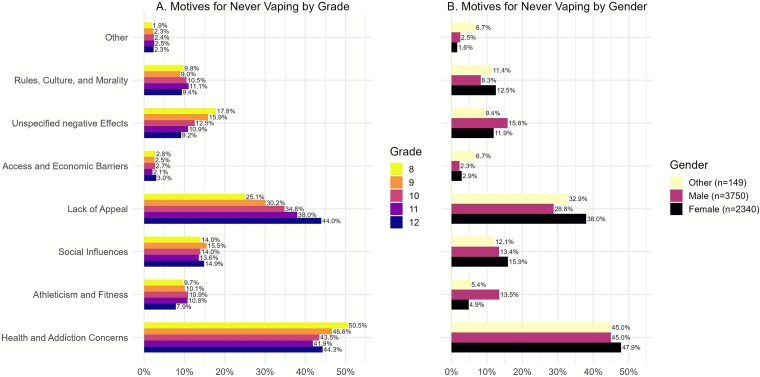
Motives for never vaping.

Among learners who reported never having vaped, health and addiction-related concerns emerged as the most cited motives for not taking up e-cigarette use, as this motive was mentioned by almost half (46.0%) of the sample (Figure 1). Representative examples of these concerns were statements such as “I don't want to become addicted (female, 14)”, “Health is wealth (male, 16)”, or “It's […] watermelon flavoured cancer (male, 14)”.

Relatedly, athleticism and physical fitness were also commonly cited as reasons for not initiating vaping, particularly among male learners. Specifically, 13.5% of boys mentioned being an athlete or wanting to stay fit as a motive, compared to approximately 5.0% of females and learners identifying with other genders (Figure 1B). Example statements were: “Because I play sport it will negatively affect me (male, 14)”, and “I dance 6 days a week at a professional level if I were to vape or smoke it would impact my dance […] (female,16)”.

A general lack of appeal was another common reason for not starting to vape, cited by 32.4% of the learners who never vaped. Responses reflected perceived lack of benefit or aversion toward vaping. For example, “I don’t see the point in doing it (female, 14)”, “I personally think vaping is stupid […] (male, 15)”, or “Its gross and smells bad (female 14)”. Notably, the salience of this motive increased with age (Figure 1A). About 25.0% of grade 8 (average age 13) versus 44.0% of Grade 12 (average age 18) learners cited it (Figure 1A). This suggests that perceptions of vaping as unappealing grow as learners mature.

The themes of social influence from friends and family; unspecified negative consequences; and rules, culture, and morality were mentioned by a smaller subset of the overall sample (10.0%–14.0%). Only a few learners (2.6%) mentioned access and economic barriers as reasons for never starting vaping (Figure 1).

### Motives for Quitting Vaping

**Figure 2:**
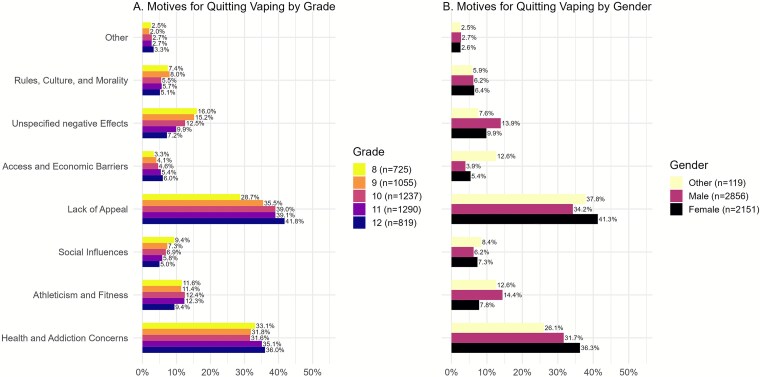
Motives for quitting vaping.

Among the sampled learners who reported that they quit vaping, lack of appeal was the most frequently mentioned motive (Figure 2); 37.3% of the learners stated motives such as: “I felt that the substance does not bring any joy to my life (male, 17)”, “[I] used a vape twice, didn’t enjoy […] (male, 17)”, and “It’s not as nice as people describe it to be (female, 15)”. Notably, older learners were more likely to cite lack of appeal than younger learners, with approximately 28.7% of Grade 8 learners and 41.8% Grade 12 learners expressing this motive for quitting vaping (Figure 2A).

Health concerns and fear of addiction were also commonly cited motivations. A total of 33.5% of former vape users cited these motivations as reasons for stopping. For example: “I decided to stop, because I don't want to destroy my lungs (female, 13)”, “I did not want to become addicted (female, 15)”, and “I stopped because I don't want black lungs (male, 14)”.

Furthermore, a small subset of learners indicated that they obtained new information that made them change their minds: “I tried it but after hearing about the future effects it will have on my health I stopped. (female 16)” or “I stopped because I learnt of the dangers of smoking and vaping (female 14)”. Athletic performance and fitness also played a role in the decision to stop. This motive was stated by 11.6% of the sample. Example statements included: “I have stopped it all because I am a sports man who needs his lungs to do well (male, 17)”, “Cause I wanted to do good in sports and therefore I couldn’t harm my lungs (female, 15)”.

Unspecified negative consequences; social influence; rules, culture, and morality; and access and economic barriers were again mentioned by a smaller part of the learners who quit (12.1%; 6.7%; 6.2% and 4.7% respectively).

## Discussion

This study draws on qualitative data from over 11 000 South African high school learners, making it one of the most extensive investigations into adolescent decisions around vaping within a global south context. Findings offer unique insights into adolescents’ self-reported motives for vaping abstinence and cessation, while highlighting variations by school grade and gender.

Health risks and fear of nicotine addiction emerged as the most prominent reasons for both never starting and stopping vaping. These findings align with reviews and population-level studies that commonly underscore health concerns as an important motive for smoking non-initiation or cessation.^10^^,^^16^^,^^17^ Closely related, athleticism and sport ambitions seem to be an additional consideration among the learners in our sample, specifically among boys.

Lack of appeal became an increasingly important reason for both not starting and stopping vaping as learners progressed through high school. This aligns with a smaller U.S. qualitative study, where adolescents cited losing interest, viewing vaping as “uncool”, and health concerns as key reasons for stopping.^18^ Vaping may lose its appeal over time due to shifting social norms and maturing personal priorities. This presents an opportunity: rather than focusing solely on risks, interventions could reinforce existing beliefs that vaping is uncool, unnecessary, or unpleasant. Certain decisions, such as stopping due to health concerns, also appear to emerge over time, possibly following negative health experiences. Additionally, students reported stopping after receiving new information, suggesting that ongoing education and health reminders can support cessation.

Extrinsic motives such as accessibility and price do not seem to be major barriers to e-cigarette use among learners in South Africa. This represents a missed opportunity given that economic measures such as higher excise taxes, and regulation banning age sales to minors have consistently been shown to reduce demand for addictive substances globally.^19^^,^^20^ In South Africa, aside from a small excise tax, the sale of e-cigarettes remains largely unregulated.

A bill to regulate e-cigarettes like tobacco has been under discussion since at least 2017. Meanwhile, the local vaping industry claims to sell only to those aged ≥18, but this is not reflected in practice. Among ≥25 000 high school learners surveyed by van Zyl-Smit et al.,^4^ 16.8% reported current e-cigarette use, indicating easy access. Stronger regulation with enforced age restrictions, tighter marketing controls, and higher taxes is urgently needed to reduce youth vaping in South Africa. As of 2025, no regulations prevent access, and vaping products are easily purchased through delivery services and local shops.

Other actionable steps to prevent vaping initiation and encourage adolescent cessation include peer-led educational campaigns and offering healthy alternatives to risky behavior. Leveraging local, relatable role models that champion a healthy lifestyle such as accomplished athletes could contribute to the success of these campaigns. Community-based organizations and the Department of Basic Education could be strategic partners in anti-vaping campaigns in the South African context.

These findings have wider relevance for countries across the global south, where weak regulation and aggressive marketing leave adolescents especially vulnerable to vaping. In several countries, young people face easy access, targeted marketing, and limited information about health risks. Strengthening enforcement of age restrictions, raising taxes, and implementing locally grounded education campaigns will be crucial to protect adolescents and curb the spread of vaping.

Although a large sample of adolescents in South Africa was compiled, responses are potentially not representative for all learners in South Africa, as selection bias toward schools with higher prevalence of vaping is possible, given the non-probability sampling strategy.^3^ The analysis identified qualitative differences in motives by gender and age, though statistical testing was not intended. Therefore, these observations should be interpreted with caution. Self-reported responses may also be subject to social desirability bias.

In conclusion, our study shows the various motives of adolescents to not initiate vaping and motivations for vape users to stop. These findings offer valuable evidence to guide the design of culturally relevant, developmentally appropriate prevention, and cessation strategies. Future research should evaluate the effectiveness of these approaches and continue to track shifts in adolescent attitudes and behaviors as vaping products evolve.

## Supplementary Material

appendix1_coding_sheet_adolescent_vaping_cessation_ntaf266

appendix2_coding_sheet_adolescent_vaping_abstinence_ntaf266

## Data Availability

The data that support the findings of this study are available on request from the corresponding author, S. Kurten. The data are not publicly available due to privacy restrictions.
