# Supplementary material for: Why Adolescents Choose to Stop or Never Start Vaping: Evidence From South Africa
Source: Nicotine Tob Res. 2025 Dec 24;28(7):1263–7. doi: 10.1093/ntr/ntaf266 (PMC13286643; doi:10.1093/ntr/ntaf266)
Supplement: appendix2_coding_sheet_adolescent_vaping_abstinence_ntaf266 [file appendix2_coding_sheet_adolescent_vaping_abstinence_ntaf266.docx]

**Codes Adolescents Vaping Abstinence**

**1. General Health concerns: (A)**

Direct health concerns (like effect on lungs) resulting from vaping are mentioned.

- Adolescents are aware of harm caused by vaping: "It damages your lungs," "I know the possible health risks," "Popcorn lungs," "Bad for you."
- Long-term health risks associated with vaping are mentioned: "Potential health implications," "Could lead to cancer," "Future health risks."
- "My body is my temple," "I take pride in my health."

**2. Addiction concerns: (A)**

Addictive elements and fear of addiction are mentioned.

- Addiction is named as a concern: "Scared to get addicted," "It’s an addictive drug," "Pointless addiction."
- Vaping as a bit habit: “it’ll just start a bad habit that I can’t get out of”
- Vaping as gateway to other drug use

**3. Athleticism and Fitness: (B)**

Sport performance is cited as the main reason to not vape.

- Negative consequence on athletic performance: "I play sports and it impacts your lungs," "I am training as a runner," "I take my sport very seriously."
- Indication that vaping is bad for athletic career: "I want to pursue my rugby career," "I want to play sports professionally."
- Negative impact on fitness: "I prefer my body in good shape."

**4. Family Socialization: (C)**

Positive influence of parents and cultural background as reasons to not vape.

- Parental guidance: "My mom showed me research," "Parents told me not to,"
- Negative example set by parents: “My mother smokes”
- Early life socialisation: “I was taught from a young age to not get into these type of things”
- "Muslim parents."

**5. Peer Socialisation: (C)**

Peers who vape are seen as negative and adolescents do not want to be associated with those.

- Avoiding peer pressure: "Peer pressure is stupid."
- Aversion against being associated with adolescents who vape: "I don’t want to be grouped with those people"
- Reputation concerns: "It would mess up my reputation," "I don’t think it’s cool."
- Changes interaction with peers: “changes how you act around people”

**6. Perceived Lack of Benefits: (D)**

Adolescents don’t see benefits in vaping.

- Pointlessness: "There’s no point in it"
- Waste of time: “It’s a waste of time
- "It doesn’t appeal to me," "I don’t see the need", “I don’t want to”, “Don’t want to try it”
- “I don’t care for it”

**7. General aversion: (D)**

Explicitly negative aversion against the practice of vaping.

- Dislike of vaping: "It smells bad," "I hate smoke," "It burns your throat."
- “I don’t like it”, “Vaping is stupid”, “It’s dumb”
- “It looks bad”, “Breath Air”

**8. Lack of Access: (E)**

Adolescents can’t access vaping products.

- “I also do not have access to such items”
- “I was never offered a vape”

**9. Unspecified negative consequences: (F)**

- “It is not good for you”
- “It’s bad for you”
- "I understand the side effects,"
- "I’ve seen the dangers," “I know the consequences”

**10. Fear of unknown negative consequences: (F)**

- “I don’t want to find out the consequences of vaping underage”
- “I do not want to use a product that I don’t know of the contents”
- “I don’t know the chemicals”, “don’t want to take the risk”

**11. Fear of sanctions / breaking of rules: (G)**

Fear of sanctions by parents/caregiver at home of adolescents

- Against rules: “Against house rules”, “It's illegal”
- Sanctions by caregiver: "My family would kill me."
- Being underage: “I am underage”

**12. Cultural, moral, and religious reasons: (G)**

- Cultural background: "It's against my culture,"
- Morals: “It’s against my believe”

**13. Economic motives: (E)**

**•** Cost concerns: "It’s a waste of money," “too expensive”

**14. Other (H):**

Other responses that are usable but can’t be clearly attributed to one of the codes.

- "My school discourages vaping"
- "I study biology,"
- “I’m not dumb” (some state that vaping is dumb/stupid. Not sure yet how to code that)

**15. Unclear/discuss**

**0. Unusable**

**Themes**

**A) Health, Fitness, and Addiction Concerns**

**General Health concerns**

**Addiction concerns**

**(B) Athleticism and Fitness**

**(C) Social Influences**

**Family Socialization**

**Peer Socialization**

**(D) Lack of Appeal**

**Perceived Lack of Benefits**

**General aversion**

**(E) Access and Economic Barriers**

**Lack of Access**

**Economic motives**

**(F) Unspecified negative Effects**

**Unspecified negative consequences**

**Fear of unknown negative consequences**

**(G) Rules, Culture, and Morality**

**Fear of sanctions / breaking of rules**

**Cultural, moral, and religious reasons**

**(H) Other**
